# 

**Published:** 1891-08-22

**Authors:** 


					246 THE HOSPITAL. Aug. 22, 1891.
NOTICE TO CORRESPONDENTS.
All MS., Letters, Books for Review, and other matters intended for the
Editor shonld be addressed The Editor, The Lodge, Porchesteb
Square,London, W.
The Editor cannot undertake to return rejected M.S., even when
?ocompaniod by stamped directed envelope.
Notice to Advertisers and Subscribers.
All Advertisements, Orders for Copies of The Hospital, and Business
Communications should be addressed to The Publisher, not to the Editor,
The Hospital, 140, Strand, W.O.
The Cost of Subscription, post free, is as followsPer week, lid.;
per quarter, Is. 8d. ; per half-year, Ss. 3d.; per annum, 6s. 6d,
ForeignPer annum, 8s. 8d.; payable, in all cases, in advance.
NOTICE.?The Index for Vol. IX.. fending1 March, 1891)
is on sale at the Office of this.i Journal, price 2?d.,
post free.
Aug. 22, 1891. , THE HOSPITAL, 247
General Charities.
[This Column is devoted to an account of work of charitable institu-
tions other than Medical Charities. The Editor will be glad to receive
reports or news of work at 140, Strand. Philanthropy should be
plainly written on the envelope.]
Lord Tennyson appeals to the public to support the1 GORDON'
BOYS' HOME, which is much in need of funds. We are sure that the
public only require to be informed of the needs of the institution, and a
hearty response to the appeal will be forthcoming.
The Treasurer to the Children's Country Holiday Fund has received a
donation of ?50 from the Clothworkers' Company; and the Fishmongers'
Company and Mercers' Company have given one hundred guineas, and
fifty guineas respectively towards removing the deficiency of ?5,000 on
current expenses of the Homes for Little Boys, Farningham and
Swanley; the Linen and Woollen Drapers* Institution has also received
one hundred guineas from the Drapers' Company.
The sum required to complete the new SEAMEN'S INSTITUTE'
Millwall Docks, is now nearly made up. Lord Brassey, the President of
t>e British and Foreign Sailors' Society has sent ?50 for the purpose.
Mr. William D. James, who has become a Vice-President of the Institu-
tion in the place of his brother, Mr. F. L. James, who was killed some
months ago by an'elephant in Africa, has sent a;similar sum, and Mr.
John Cory has promised ?100 conditionally that the remaining ?300 is
given.
Miss Florence Nevill is anxious to increase the supply of books for the
blind, and with a view to doing this has established THE BRAIIiIiE
BOOK SOCIETY, the headquarters of whioh are at S, Victoria
Mansions, Grand Avenue, West Brighton. She appeals for help to enable
her to place the Society on a firm basis, and asks for the value of one
Braille book a-year from anyone who is willing and able to further her
excellent undertakir g. The account of the Braille book will be forwarded
to the donor, who is asked to give the value of a book of one pound or
fifteen shillings, ten shillings or five shillings. The object of the Society
is to buy the books written by the blind writers of the British and
Foreign Blind Association, and to give them away to asylums and
libraries for the blind.
Lord Monkswell, as Chairman, and Miss Octavia Hill, as Treasurer of
THE KYEIE SOCIETY, appeal to the public for help to carry on
their work. For some years past the Society has been doing useful
work as a purveyor of musio' to the people. In summer bands are en.
gaged to play in different publio gardens situated in the poorer districts
of London; and in winter oratorio concerts are given by the Society's
choir, and lighter entertainments are organised under the direction of a
musical sub-committee. The plaoes chosen by the Committee for their
operations are invariablyithose which furnish audiences most largely
composed of the really poor, and the work done by the Society is not
only one whioh gives pleasure to many who have few opportunities for
any brightness in their lives, but is one of an elevating nature. Admis-
sion is tree always, so that the Society is compelled to appeal to the
wealthier portion of the London inhabitants to help them in their
labours, the popularity of which is evinced by the ever-increasing appeals
ior the Society's services. Donations can be sent to the Hon. General
Secretaries, at the office, Manchester Street, W., or to the Society's
account with the Branch of the National Provincial Bank'of England in
Baker Street. ' "
The holiday season is now in fnll swing for all members of society, and
even rin??* and paupers are allowed a short respite from the monotonous
routine o eir aily existence. Among those who are eagerly looking
fora change ara many of the working boys of London, and on their be-
half ^"ington and other members of the Committee of
THE SEASIDE CAMP POB> WORKING BOYS appeal.
Though, as they point out, the aims and results of the work are now
well known, both to working toys all over London, and to those who
take an interest m them, the aims of no society are so well known as to
enable the managers to dispense with requests for aid from time to time.
With replenished funds the Committee hope by the end of the summer
season to have received more than 1,500 boys at Deal, where this year
they have pitched twenty tents and two marquees on the sand hills. In
the camp, whichis again under the command of Colonel Farquharson, 160
boys a week are being received. They wear a sailor uniform, have
regular drill and exercise, are taught to swim and play cricket,
and are required to make themselves generally useful. The expense!
of the holiday are not wholly borne by the Committee, as eaoh lad pays
3s. 6i, for one week, or 6s. for a fortnight; but the greater part of the
expenditure arising from railway fares, &c., has to be defrayed from
subscriptions. This year the Sooiety has received valuable help from
the publio schools. Eton, Harrow, Haileybury, Bradfield, and other
schools have sent offertories from the boys' pocket money for the
benefit of their less fortunate contemporaries, and several Rugby
masters are in the camp teaching vhe lads how pub-ic school-boys ?' live
and play and utilise leisure " Though the Committee are in friendly
allianoe with the variouB Children's Holiday Funds, their special work
is to take bojs who are too old for such funds and t o poor to take
themselves. It is hardly necessary to point out what lasting good
accrues to the boys from the camp discipline, sport, sea-bathing, and
healthy life.
Scraps and Gleanincs.
The Fleetwood Cottage Hospital was opened on the 8th nit.
The subscriptions towards the Cottage Hospital for West Fife already
amount to ?6,347.
The Blackpool Hospital scheme is rapidly progressing. ?337 has
been definitely promised, besides a sum of over ?1,200 for building pur-
poses alone.
The Glothworkerh' Company have sent ?50 to the Hon. Alfred Lyttel-
ton for the Children's Country Holidays Fund, the subscriptions to
which hare fallen off this year.
It has been decided to make several alterations in the internal
arrangements of the Inverness Infirmary before appointing a new
Matron in place of Miss Falconer, lately deceased.
The Friendly Societies of Littlehampton and district held an amalga-
mated demonstration on the 10th ult., when the sum of ?25 was col-
lected and handed over to the Chichester Infirmary.
The Meath Hospital and County Dublin Infirmary congratulates itself
that its expenditure under every head is lower than in any other general
hospital participating in the Dublin Hospital Sunday Fund.
A noisy and disorderly meeting of the QueenBtown Dispensary Com-
mittee was held on the 6th in&t. for the purpose of electing a medioal
officer. The meeting adjourned, no decision having been arrived at.
The Committee appointed to consider the proposed extensions at the
Beverley Infirmary are already in difficulties. At the end of their first
meeting, they came to the conclusion they did not know what they
wanted, but are going to try aid find out before they meet again.
At the quarterly meeting of thej Newcastle Infirmary the subject of
the proposed new infirmary was brought forward and a motion propos-
ing that a subscription liBt for the purpose should be started, was carried.
Professor Adam Kiewicz, of Cracow, has, says Dalziel, just com-
municated to the Academy of Sciences the result of a series of success-
ful experiments whioh he has been makirg with " Cancroin," the new
cure for cancer.
Mdlle. Nicoixe, the well-known Lady Superintendent of the Hos-
pital Salpetriere, France, has resigned her post after a service of forty-
two yeais' duration. Two years ago she was decorated with the Cross of
the Lsgion of Honour.
The ninety-second annual report of the Sunderland Infirmary shows
that the work has been carried out in an efficient manner, that there was
an increase in the number of patients and that the hospital was in a
satisfactory financial position.
An example of the dangers which hospital nurses are subjeot to is
given in the Gflobe. A nurse at the Bradford Infirmary has been bitten
by a patient suffering from hydrophobia. She ha3 been sent to Paris to
undergo the Pasteur treatment.
Arrangements for the laying of the foundation of the new hospital
at Dundee are being made on a very large scale. A very enthusiastic
demonstration is expected, in spite of the fact that the movement has
met with opposition from some of the working classes.
A happy suggestion is made by a gentleman at Cottingham to those
who with to brighten the lot of patients in our hospitals. He proposes
that in place of the flowers sent trom flower services, that little bags of
lavender are very acceptable, and the enjoyment is more lasting.
At the annual meeting of the Governors of the West Bromwich Dis-
trict Hospital, it was shown that the in-patients had increased by 103.
The income has increased by ?540, but the expanditura still shows a
deficit on the accounts, and the hospital is nearly ?1,000 in debt.
A movement is on foot among the Surrey tenantB of the Duke of
Northumberland to commemorate the late Duchess by providing a
number of cottage3 for the aged poor in the district, in whose welfare
her Grace always evinced a warm interest. A similar idea is being
entertained as Alnwiok.
Favoured with a fi ae evening, the second annual open-air service of
sacred music was held at Stainbro' last Sunday, in aid of the funds of the
Barneley Beckett Ho'pital, and proved quite a success. The attendance
was large, the programme a good one and admirably rendered, and a
satisfactory sum was realised for the Hospital.
The Castle Donington Convalescent Home, one of the three institu-
tions of the kind benefiting by Colonel Jeeley's munificent gift of
?10,000, was formally opened by Lady Belper on the 30th ult. The
building consists of three storeys, is most commodious, and stands in
spacious grounds, which are beautifully laid out.
At the sixth annual meeting of the subscribers to the Totnes Cottage
Hospital the Committee congratulated the subscribers and supporters
that the sixth year of the existence of such a useful institution had been
to successful as its well-wishers could desire. A good work had been
done, and the financial condition of the Hospital was also satisfactory.
On the 5 h inst. t/ e Duke and Duchess of Fife pa d a visit to the
Chalmers Hospital, Banff, which is open to pat <nts fr >m the whole ot
Banffshire. The J.<uke and Duchess ex Dressed fatisfact on with all the
arrangements, and Her Royal Highness his sent to Mrs. uray, the
Matron, photographs of herself and the Dnke.
The Preston Union certainly manage their nursing arrangements
very straneely. They are in connection ?ath a nursing association, yet
for sometime they have failed to supply the vacanoie3 on the staff, and
one nurse now has the charge of eighty patients night and day. She
has s^me untrained assistance, and the serious cases are reported to bo
very few.
It is proposed to build a cottage hospital at Nuneaton. It is also
learned that the lord of the manor, Mr. Tomlinaon, his given a suitable
site, and a sum has been promised which will probably enable the
building to bo oomme^ced at once. Under the circumstances the
:Bed worth Committee have dropped the idea of a street collection in aid
of the institution.
The erection of the new Sick Hospital at Dundfe has entailed the
addition of a penny to the poor assessment imposed by the Dundee
Combination. Ttie aeconnts of the Board for the past year showed a
favourable balance of ?200. In the estimates for the current year there
is an increase of between ?300 and ?409 in the expenditure for ordinary
purposes of administration.
Atjg. 22, 1891. THE HOSPITAL.
The Student of Medicine.
[All communications for this Supplement should be addressed to The Editob of The Hospital, at 140, Strand, with the words "Students-
Column, written in the left hand top corner of the envelope.] *
APPOINTMENTS.
At the Great Northern Central Hospital, Hollo way Road,
^ordon Calthorp, M.B., B.C., Cantab., has been appointed house
Physician. At the Hospital for Sick Children, Great Ormond
street, Francis George Penrose, M.D., M.R.C.P., has been ap-
pointed assistant physiciaD. At the Royal Albert Edward In-
orinary and Dispensary, Wigan, Weldon C. Carter, M.D. Lond.,
^?R.C.S., has been appointed senior house surgeon. At the
^erbyshire Royal Infirmary, J. Winter Dryland, M.R.C.S.,
~-R.C.P., has been appointed resident assistant house surgeon.
the Stanley Hospital, Liverpool, J. Guest Gomall, M.R.C.S.,
~*R.C.P., B.A. Cantab., has been appointed house surgeon. At
General Infirmary, Wrexham, G. A. Lancashire, M.R.C.S.,
^?R.C.P., has been appointed house surgeon.
VACANCIES.
The following vacancies are announced this week, the amount of
"Wary in each case being given after the name of the institution:
Resident Medical Officers.
Coventry and Warwickshire Hospital, House Surgeon?Salary ?103, and
wWard, &c.
wderjfield Infirmary, Junior House Sargeon?Salary ?40, and
\r ardf &c.
Chester Royal Infirmary. Resident Surgical Officer?Salary"?150.
j8nd board, &c.
^'herham Hospital, Resident House Surgeon?Salary ?100, and
? ward, &c.
?yal Portsmouth, Portsea, and Gospoit Hospital, Portsmouth,"Assist-
House Surgeon?Board, &c.
Victoria Hospital, Bournemouth, House Surgeon and Secretary?
"alary ?100, and board, &c.
g . Non-Resident Medical Officers.
nij^ton and Hove Lying- in Institution, Honorary Surgeon.
vueen's College, Birmingham, Leoturer on Operative Surgery.
?gapore, Straits Settlements?Health Officer?Salary 800 dols. per
^snsem, and house allowance.
PASS LISTS.
?oyal Colleges of Physicians and Surgeons of England.
tM i0 followinK gentlemen, havincr passed the necessary examinations
i having conformed to the bye-laws and regulations have been, at a
meeting rf the 0illoge of Physicians, admitted licentiates of the College:
Edward Henry Alexander, student of Edinburgh University ; Winslow
Anderson, of California; Bennett Harvey Andrew, of King's College
Hospital; Henry Brunton Angus, of Newcastle-on-Tyne; Henry
Stewart Archdall, of Guy's Hospital; Francis Arthur, of St. Bartholo-
mew's Hospital; John Francis Atkins, of Birmingham , William Tilling -
hastAtwool, of St. Mary's Hospital; Henry John Francis Badcock, of
Charing-cross Hospital; Thomas Bamford, of University College Hos-
pital ; Alfred Banks, of St. Thomas* i Hospital; Leonard Stewart Barnes,
of St. Bartholomew's Hospital ; George Harold Comyns Berkeley, of
Middlesex Hospital; Arthur Campbell Black, of University College
Hospital; Edward Eldridge Blomfield, of London Hospital; Francis : I!,
Scencer Bond, of St. Mary's Hospital; John Francis Harpin iBroadbent,
of St.Mary's Hospital; William James Broadhurst, of University College ;
Tom Stanbury Brook, of St. Bartholomew's Hospital; John Ridley Bur-
nett. of Edinburgh University and Middlesex Hospital; William Edward
Burton, of Liverpool and London Hospital; Thomas Spry Byass, of Uni-
versity College Hospital; Georero Carolin.of University College Hospital;
Thomas Moravian Cartsr, of Bristol; Albert John Chalmers, of Liver- 11
pool and London Hospital; Claude Churchill Chidell, of University
College Hospital; Harry Ward Clarke, of Charing Cross Hospital;
William Newlands Clemmey, of Liverpool; Thomas Treffry Cockill, of
Middlesex Hospital; Thomas Arthur Collinson, of King's College Hos-
pital; Maurioe Oraig, of Guy's Hospital; Frederick James Abercrombie I
Dalton, of St. Bartholomew's Hospital; Fryce Edward Davies, of :|l'
Liverpool; Thomas BeDjamin Philip Davies, of Guy's Hospital; Arthur
Dixon, of Manchester; Edward Miall Dobinson, of Guy's Hospital; ,
Norman Dowling, of St. Bartholomew's Hospital; Herbert Annesley
Eccles, of St. Bartholomew's Hospital; John William Emmett, of Guy's
Hospital; Walter Pemberton Pooks, of Cambridge and St. Thomas's
Hospital; Harvey Francis, of St. Mary's Hospital; John Ernest 3ulli-
van Frazer, of St. Bartholomew's Hospital; John Reginald Fuller, of
St. Mary's Hospital; Arthur Gladstone Gine, of St. Bartholomew's
Hospital; Sydney Dalton Graham, of St. George's Hospital; Henry
Greaves, of St. Thomas's Hospital; Patrick Balfour Haig, of Edin-
burgh University; Percival Seymour Harris, of Middlesex Hospital; Jlj
Claude Haye, of 8t. Bartholomew's Hospital; John Heminswiy, of St. ! ;
Thomas's Hospital; George Hern, of Middlesex Hospital; Rabert George
Hogarth, of Sc. Bartholomew's Hospital; Charles Denton Holmes, of
Liverpool and Edinburgh University; Otto Leonard Hoist,' of St. Bar-
tholomew's Hospital; Hubert Lindsay Curling-Hope, of Middlesex
Hospital; Robert Dunmore Hotchkis, of Newcastle-on-Tyne and St.
Bartholomew's Hospital; Joseph George Hulbert, of St. Bartholomew's I ,|
Hospital and Cambridge University; Arthur Dumville Humphry, of St.
Bartholomew's HosDital; Frank Ernest Ingall, of London Hospital;
THE HOSPITAL,
Aug. 22, 1891.
THE STUDENT OP MEDICIWE-fcontmued).
Rupert Jamesf, of University College Hospital; John William Frank
Jewell, of Guy's Hospital; Harry Oswin Johneon, of London Hospital;
Dudley Oater Johnston, of Gharing-croea Hospital, Alfred Stanley Jones,
of University College Hospital; George Jones, of London Hospital and
Oxford University; Clxarle3 Ernest Mackenzie Kelly, o! Manchester;
William Willoughby Kennedy, of St. Bartholomew's Hospital; Ernest Mil-
ward Knott, of St. Mary's Hospital; Harry Angell Lane, of London Hos-
pital ; Arthur Edward Anthony Lathbury, of Sc. Bartholomew's Hospital;
John Lawrence, of St. Bartholomew's Hospittl; Forest Bertram Leeder,
r>f University College Hogpital; Sidney Herbert Lucy, of Bristol; Hans
Hamilton Mack, of London Hospital; Charles Edward Alexander M'Leod,
of Westminster Hospital; Evan Cameron MaoLeod, of Westminster Hos-
pital; James MaoNeal. of London Hospital; Arthur Ernest Madge, of
London Hospital; William Harvey Maidlor/, of St. Bartholomew's Hos-
pital; Arthur Manknell, of Leeds; Cyril Darby Marson, ot Birmingham ;
Walter Cyril Mayo, of Leeds ; William Ernest Miles, of St. Bartholo-
mew's Hospital; Herbert Henry Mills, of Westminster Hospital;
Edward James Fleetwood Moore, of London Hospital; Vincent Moxey,
of Bristol; Robert Douglas Muir, of Charing Cross Hospital ; John
Mulvany, of London Hospital; John Bassett Naden, of Manchester;
Edward Albert Nathan, of St. Mary's Hospital; Nevell Edmund Norway,
of St. Mary's Hospital; Orisadipe Obasa of Ikija (Georce Samuel Stone
Smith), of St. Thomas's Hospital; John Orr. of Edinburgh University;
Thomas George Ouston, of Leeds; Thomas Edward Allaway Pearman,
of London Hospital; Albert Edward Duncan Ralph Peters, of St. Bar-
tholomew's Hospital; William Prior Purvis, of St. Thomas's Hospital;
Harold Meredith Riohards, of University College Hospital; William
Ernest Rielly, of University College; Francis William Rix, of Westmin-
ster Hospital; Herbert John Ropei, of Leeds ; Charles Edward Salter, of
Guy's Hospital; George Severs, of St. Mary's Hospital; Joseph Sef ton
Sewill.of Guy's Hospital; Percy Sharp,of King's College Hospit al; Francis
Bentley Shaw, of Middlesex Hospital; Samuel Shore-Smith, of St.
Bartholomew's Hospital; John Smale, of St. Bartholomew's Hospital;
David Wright Luther Soutter, of King's College Hospital; Mansfield
Knox Soutter, of King's College Hospital; Henry Newport Stephens,
of Manchester; Frederick John Orchard Stephenson, of St. Mary's
Hospital; William Mitchell Stevens, of University College Hospital;
Henry Archibald Stonham, of Westminster Hospital; John Odery Symes,
of St. Mary's Hospital; Frederic Henry Arthur Taylor, of Charing
Cross Hospital; David Owen Thomas, of St. Bartholomew's Hospital
and Indiana; Charles Thompson, of Charing Cross Hospital; George
Thornton, of Edinburgh University; John Joseph Waddelow, of King's
College Hospital; James Philip Stephens Ward, of St. Bartholomew's
Hospital; George Alexander Watson, of University College Hospital;
Henry Meilor Woaver-Bridgmau, of St. George's Hospital; Percy Law-
rance Webster, of King's College Hospital; William Henry Willans, of
London Hospital; David Lewis Williams, of London Hospital: Colston
Wintle, of Bristolj Ernest Wells Witham, of Westminster Hospital;
Reginald Carmiohael Worsley, of University College Hospital; acfi
Francis James "Worth, of St. Mary's Hospital.
University of London.
PRELIMINARY SCIENTIFIC (M.B.) EXAMINATION, JULY, 1891.
Entire Examination.?First Division: 0. W. Alford, Epsom College;
W. B. Anderton, University School, Southport, and Owens College; *S,
B. Atkinson, St. Bartholomew's Hospital and private study; *F. A.
Bainbridge, The Loys, Oimbridge; H. L. Barnard, private study and
University Tutorial College; *W. R. Battye,University College, Bristol,
Clifton Laboratory, and private tuition; R. H. Birtwell, B.A., Owens
College; J. H. Bodman, University College, Bristol, and private study ;
J. A. O. Briggs, University College, Nottingham, ardQaeen Elizabeth's
School, Mansfield; T. R. H. Bucknall, Mason College; T. Chave, St.
Bartholomew's Hospital; M. W. Coleman, St.Bartholomew's Hospital;
H. H. Greenwood, the Yorkshire College; *W. S. Henderson, University
College, Liverpool; *A. C. Hill, St. Bartholomew's Hospital; *G. H. J?
Hooper, private study and Charing Cross Hospital; T. J. Horder, St.
Bartholomew's Hospital; *8. P. Huggins, St. Bartholomew's Hospital
and Birkbeck Institute; *J. 0. H. Leicester, University College and
private tuition and study; J. P. Maxwell, St. Bartholomew's Hosoital
and Birkbeck Institute ; A. A. Montague, St. Thomas's Hospital: S. D.
Soott, St. Bartholomew's, Birkbeck, and Mercers' School; *A. J. Smith,
"Westminster and Middlesex Hospitals; Florence A. Stoney, Royal Col-
lege of Science, Dublin, and private study; H. Sugden, St. Mary's
Hospital; *F. G. Thomson. Epsom College; L. Tong, Owens
College; W. B. L. Trotter, University College; Ethel M.
Vaughan, University College; "W. Wilkins, Epsom College; H.
Williamson, The Leys and St. John's Colleges. Cambridge.
Seconi Division: A. Ali, St. Bartholomew's Hospital; R. H. Ashwin,
Dulwich College; F. H. Atkinson, Epsom College; G. F. Bergin, Uni-
versity College, Bristol, and private study; S. L. Box. St. Bartholo-
mew's Hospital; *H. <VT. Oastledine, private study ; *F. 0. W. Clifford,
King's College; A. S. Cobblediok, University College, Bristol; D. H. F.
Oowin, University College; H. P. Ferraby, Guy's Hospital; *R. J. Fox,
Owens College; *L. F. Giblin, University College; F. "W. Goldie. Uni-
versity College; O. B. Goldschmidt, Owens College ; H. A. Good, King's
College; *0. B. Goring, University College; 8. Gross, the Yorkshire
College ; P. H. Haylett, Guy's Hospital; *A. Heath, University College
Nottingham ; A. Holroyde. the Yorkshire College; J. Howell. Guy's
Hospital; H. Innes, University College; P. Levick, Epsom College;
R. E. W. Mackenzie, Heriot Watt College of Surgery, Edinburgh; A. J.
Martineau, University College and private study; J. H. Murray, Epsom
College; *G. H. Pethy bridge, University College, Aberystwith: G.J.
E. Pitman, Clifton and University Colleges; H. S. Pyne, private study
and tuition; J. W. F. Rait. University College and private study ; *A.
0. Rose, Mason College; *J. D. Russell, University College, Birkbeck
Institute, and City o? London School; J. M. Schaub, King's College ;
? ;f
Aug. 22, 1891. THE HOSPITAL.
J"
THE STUDENT OF MEDICINE-(continued).
E 't?' Stevenson, St. Mary's Hospital; D. B. Todd, Gay's Hospital; 0.
niV.i m^le? University College; A. B. Tacker, St. Bartholomew's Hos-
Bno -i. r- Tucker, London Hospital; P. T. Waldron, London
jr ?p!JaM S. R. "Walker, Epsom College; A. E. Walter, Middlesex
spital; W. "Wrangham, St. Bartholomew's Hospital.
Ki???0UES Candidates Recommended fob a Pass.?G. H. Lansdown,
College; J. P. Porter, the Yorkshire College; E. Thomas,
jLlversity College , Aberyetwitb, an-1 private study.
Tai?EiIISTKY AND Expekimental Physics.?R. Balderson, Merchant
nft?ior?' School; W. B. Bell, King'slCollege ; S. H. Berry, Kent College,
Wiry; +A- H. Bygott, Grammar t-chool and School of Science,
DitTi ' ancl Qneen's, Birmingham; 0. E. Durrant, St. Thomas's Hos-
FiliV-a?^ Birkbeck Institute; +W. Datton, Owens College; Jane H.
Hn f Andesron's College, Glasgow, and University College; P. S.
str.? ? Private study; tA. D. Ketchen, University College and private
jr;j,y> tW. M. McDonald, St. Bartholomew's Hospital; fW. R. S.
Private Btuay; W. G. Mortimer, London Hospital; tO. H.
p H?ray, Guy's Hospital and private tuition; W. L. Odell, private study ;
Ta?i ?ea'> Durham College of Soience; P. 0. Shrubsall, Merchant
Brio \LS School and University Tutorial College; +D. L. Smith, Guy's
tH t> ' Smith, Owens College; J. P. Swift, Pirth College;
Win Walker, King's College and Birkbeck Institute; R. Water-
AK> e'Private study and Firth College; tS. Whichor, University Collega
_ rystwith and Guy's Hospital.
?\y '0L?Gt\?.Tamfetji N. Bahadhurji, University College; H. C. Barlow,
titirel^on School, Leicester; J, A. P. Barnes, St. Bartholomew's Hos-
Bai-ti! ? W* Bincko?, Birkbeck Institution; W. P. Y. Bonney, St.
Bital w's Hospital; P. W. Brig3tocke, St. Bartholomew's Hos-
me?'5 ^'berine M. Campbell, private study; tH. Oardin, St. Bartholo-
BtJ 8 Hospital; C. J. Coleman, Mason College and private tuition and
5 iy > E. H. Coleman, Mason College and private tuition and study;
5'eh P00Per? London Hospital; O. G. Dart, University College; Alice
.m? Bedford College, London, and private study; M. Dixon,
UiPlJ^rsity College; G. E. Dodson, St. Bartholomew's Hospital and
St ,L~Gck Institution; Mary B. Douie, University College ; fB. Dyball,
En'ci 518'8 Hospital; A. B. Fry, University College; E. J. Gaman,
8t S?1 College; fP. H. Gervis, St. Thomas's Hospital; W. A. Golby,
fia^riholomew's Hospital; H. A. Giinther, University College; tO.
gj ^s> School of Science and Art, Newcastle-on-Tyne ; H. C. Harrison,
S'rjP^tholomew's Hospital; Charlotte E. Hull, University College;
Jv^' Hyde, University College; H. 0. Jackson, University College ;
Caprine R. Jay, Bedford College, London; Sarah Kaye, University
e^.ei Liverpool; H. Leader, Pirth College and Guy's Hospital;
Bt'udv leathern, University College, Liverpool, and private
Q j'i P. A. Leete, Cooper's Hill College ai>d private study;
Sart*> evick, private study and Birkbeck Institution; B. Lewitt, St.
ptj^ 8 Hospital; 0. D. Lindsey, St. Mary's Hospital; t E, E. Lloyd,
^ study; + J. T. Marsden, private study and Owens College; t J.
Moore, Gay's Hospital and private study; H. Mnndy, University
Tutorial College; Marion I. Newbigin, Heriot Watt College, Edinburgh;
J. P. Northcjtt, University College; Major B. Oliver, University
College; A. W. Oxley, University College; tE. L. Perry, St. Paul's
School and St. Thomas's Hospital; P. Phillips. University College,
Cardiff; J. J. Pierce, University College, Aberystwith, and private
study; Pritchard, Owens College and private study; 0. Riviere,
University College; W. H. Itaache, St. Bartholomew's Hospital; Claude
Bundle, private study; Annie S. D. Russell, Girton and University-
Colleges; P. A. St. John, St. Mary's Hospital; P. G. Silley. St. Paul's
School; S.P.Smith, St. Bartholomew's Hospital and private study;
0. L. A. Stievenard, University College; P. R. Sutton, Mason College ;
J. H. Tallent, University College ani private tuition; Elinor A. N.
Twigg, Mason College , t 0. R. Watson, St. George's Hospital and private
study; tH. 0. Watt, University College, Liverpool; Clarence B.
Whitehead, Wygereston School, Leicester; R. H. Wilshaw, Owens
College; H. E. Wise, Middlesex Hospital; W. 0. Wood, St. Mary's
Hospital.
Two Subjects op the Examination (under former regulations).^ i!
?J.J. L. Macfarlane (0., P.), University College; W. 0. Rowlands
(P., B.), University of Edinburgh.
One Subject op the Examination (under former regulations) .tH.?
J. A. Beloher (B), Mason College and private study and|tuition ; H. S.
Dobie (B.), Owens College and private study; S. J. H Eastwick-Pield
(B.), University College and private study and tuition; A. St. L. Pagan
(B.), London Hospital and private study; J. M. Stewart (3.), private
study.
* These candidates'have also passed in the Mathematics of the Inter-
mediate Examination in Science, and have thus become admissible to. the II
B.Sc. Examination.
t Those candidate* have now completed the examination. i
IT The Bubjects taken up by these candidates are indicated by initials 1
after the names,?0. =' Chemistry; P. = Physics ; B. = Biology.
INTERMEDIATE EXAMINATION IN SCIENCE, 1891.
Pirst Division.?G. E. Allan, University of Glasgow and private
study; H. S. Allen, Kingswood School; P. J. Bancroft, University
Tutorial College and Birkbeck Institution; E. Barraclough, West-
minster Training College and Royal College of Science; M D. Blunt,
University Tutorial College and Birkbeck Institution; J. Brown,
private study; E. G. Bryant, B.A., private study; E. J. Burrell,
Borough Road College, People's Palace, and private study; W. G. S.
Constable, B.A., University College, Nottingham, and private study;
J. Crichton, B.A., University Tutorial College ; S. J. Farthing, Birk-
beck Institution and private study; E. Green, St. Thomas Charterhouse,
Birkbeck Institution, and private study; J. A. Harrison, Mason
College; A. B. Hewart, University College, Aberystwith ; M. E. Hicks,
Westfield College, Hampstead ; H. Holbeche, Royal College of Science
THE HOSPITAL.
Aug. 22, 1891.
THE STUDENT OP MEDICINE-fcontmued).
?
? 11
I III I I 1
1II
and private study; G. D. Lander, Camberwell Grammar School and
Guy's Hospital; J. Lang, Veterinary and Anderson's College, Glasgow,
and private tuition; Fanny Lowater, University College, Nottingham ;
T. Luxton, B.A., private study and tuition; P. Macaulay, B.A.,
Borough Road College and private study; L. S. Marks, Mason College
and private study; J. Mill?, B.A... University College, Nottingham, and
private study; A. Morris, private' study; J. Orchard, Free Library,
Wolverhampton, and Mason College ; H. L. Parry, B.A., private study
and tuition; A. F. Ravenshear, private study and University Tutorial
College; T. K. Rose, private study ; W. Soudamore, Royal College of
Science, Fitzwilliam House, Cambridge, and private study ; El'iaL.Selby
University College, Bangor; J. C. Walton, B.A., Mason College,
Midland Institute, and Saltley College; Phoebe Ward ell, Royal Hollowav
College; Williams,0., University Colleges, Cardiff and Aberystwith; J.
W. Young, B.A., Royal College of Science, Yorkshire College, an a private
study.
Second Division.?J. B. Ashcroft, University College, Nottingham,
and private study; Sophia L. Beanland, the Yorkshire College ; Eliza-
beth H. Beard, Royal Holloway College; Jennie Beckingsale, Chelten-
ham Public Day School; S. A. Bennett, Queen's College, Cambridge;
J. H. Brown, the Yorkshire College and private study; F. B. Carter,
Universily College; Margaret Davies, University College, Aberystwith ;
W. N. Drew, Royal College of Science and private tuition; J. L. Forbes,
Birkbeck Institution; T. M. Gabbert, B.A., private study; E. S.
Goodrich, University College; D. A. Gracey, Birkbeck Institution and
private study: W. A. Greaves, private study; J. W. Haine, Birkbeck
Institution; G: M. Handley, Owens College and private study and
tuition ; D. Hughes, B.A., University Colleg*, Bangor ; H. Jarratt, pri-
vate study; J. S. Kearns, Dnlwich College; J. Lewis, private study;
W. J, E. Lupton, New College, Oxford, and private study; M. Papar-
ritor, Birkbeck Institution, University College, and private tuition;
J. A. Ratcliffe, B.A., Stonyhurst College; R. R. Raymer, Birkbeck In-
stitution ; G. Robb, Westminster Training College and private study ;
W. W. Saunders, &c., B.A., Brighton Sohool of Science and Art and pri-
vate study ; Rose Stern, Mason College; C. Thompson, Mason College,
Dudley and Midland Institute, and private study ; M. W. Travers, Uni-
versity College; R T. Williams, B.A., University College, Bangor;
Mary Woon, Bedford College, London.
Honoues Candidates Recommended fob a Pass.?R. M. Caven,
University College, Nottingham ; E. K. Jones, Christ's College, Cam-
bridge; J. H. Sinclair, King's College and private study; 0. H. H.
Walker, University College, Oxford, and private stady.
INTERMEDIATE EXAMINATION IN MEDICINE, JULY, 1891.
Entire Examination.?First Division.?Fanny Armitage, London
Sohool of Medicine for Women; H. Davies, University College; B. F.
Harwood, University College; A. W. Kirkpatrick-Picard, University
College; E. Knight, Owens College; G. E. Manning, Guy's Hospital;
A. P. Nuttall, University College; J. Unsworth, University College,
Liverpool. Second, Division.?W. F. Blewitt, Queen's College Bir-
mingham; B. Oolljer, St. Bartholemew's Hospital; F. N. Cookson,
Middlesex Hospital; I. Costa, University College ; E. H. Drake, Gay,8
Hospital, G. L. Eastes, Guy's Hospital; 8. E. Gill, St. Bartholomew s
Hospital; W. J Gillespie, B A., St. Bartholomew's Hospital; 0. P*
Harris. London Hospital; T. Harrison, University College; Boss?
Anne Hngho% London School of Medioine for Women : Harriet Edi'J1
F. Knight. London School of Medicine for Women ; F. W. Lewis, Sj-
Mary's Hospital; H. W. Lyle, King's College; W. A. Marris, Queens
College Birmingham ; E. Mi!-kin, St. Thomas's Hospital, E. J. Morgan-
Si". Mary's Hospital; 0. Oldfield, The Yorkshire College; F. E, Bock?
Middlesex Hospital; E. J. Smyth, B.Sc., University College; E.
Scink, The Yorkshire College; F. T. Travers. University College; *?
McKinneU C. Wilmot, Guy's Hospital.
Excluding Physiology.?First Division: S.Cornish, St. Bartholo-
mew's Hospital; 0. H. Drake, St. Bartholomew's Hospital; T. B.
Flint, Owens College; S. F. Uibba, St. Bartholomew's Hospital: A. 0*
Qurney, St. Bartholomew's Hospital. Second Division : F. L. Blenkin-
sop, University College ; W. E. DeKort6, Gay's Hospital; W. E. L?e?
St. Bartholomew's Hospital; W. H. Pollard, St. Bartholomew8
Hospital. f
Physiology Only.?First Division: A. J. Edge, St. Bartholomew3
Hospital; P. 8. Eve, University College. Second Division : E.
Aiams. Gny's Hospital; A. P. Allan, Guy's Hospital; H. K. Birley?
Owens College; 0. F. Cross, King's College; F. Hazell, Gny's Hospital S
0, M. Rhodes, St. Mary's Hospital; E. Sjy, King's College.
British Medical Service,
The following is the list of Surgeons onprob ition who were successful
at the recentLondon University examinations. Thefinal position of thes0
gentlemen are determined by the marks gained in London added to
those trained at Netley, and the combined numbers are aOcordingly sho Wfl
in the list as follows : ?
?Porter, F. J. W....
Robinson, O. L....
tStalfcaartt, 0. E. G,
Gibbard.T. W. ...
Healy. 0. J. ...
Bnrtchaell. C. H.
Buist, H. J. M. ...
Stanistreet, G. B.
Brown, P. J.
Hardy, W. E. ...
Brogden, J. E. ...
Marks.
59-n
5370
5235
5160
4992
4975
4970
4930
4910
4909
4777
Tate, G. "W.
Faichnie, N.
Lenehan, T. J,...
McDowell, P. ...
Begbie, P. W. ...
MacOarthy, J. A. O.
Austin, J H. E.
Dnggan, 0. W. ...
Gerrard, J. J. ...
Jameson, J. 0. ...
Grained the Herbert Prizq of ?20, with the Montifi re Medal and
Prize of 20 guineas, and with the De Ohanmont Prize in Hygiene,
t Gained the Montefiore Second Prize.

				

